# Supplementary material for: Patterns of Intron Gain and Loss in Fungi
Source: PLoS Biol. 2004 Nov 30;2(12):e422. doi: 10.1371/journal.pbio.0020422 (PMC532390; doi:10.1371/journal.pbio.0020422)
Supplement: Table S1 — Also available at http://genes.mit.edu/NielsenEtAl/. (4.3 MB ZIP). [file pbio.0020422.st001.zip › NielsenEtAl/html/145.html]

AN0239.1.NCU00671.1.MG02823.1.FG00191.1


```
 CLUSTAL W (1.82) Multiple Sequence Alignments - Introns Inserted


Sequence 1: NCU00671.1	2662 aa
Sequence 2: MG02823.1	2650 aa
Sequence 3: FG00191.1	2825 aa
Sequence 4: AN0239.1	2491 aa
Alignment Length: 2963 aa
Number Identitical Residues: 845 aa
Alignment Score (without introns) 50521


MG02823.1 	------------------------------------------------------------
NCU00671.1	------------------------------------------------------------
FG00191.1 	MPYPRHDFDIQVSWEPKKESPLVWIDKNSDFYKKTGIYMYSVEQNDYAYWYTYEIRIHTD
AN0239.1  	------------------------------------------------------------
          	                                                            

MG02823.1 	------------------------------------------~-----------------
NCU00671.1	------------------------------------------~-----------------
FG00191.1 	DPYAYTFYDEEGDSYDLTVNLPKFSASTHDVNYNSNRPKIVR0TSISAPECFALWHQRIA
AN0239.1  	------------------------------------------~-----------------
          	                                                            

MG02823.1 	--------------------------------------------------------MATL
NCU00671.1	-----------------------------------------------------------M
FG00191.1 	SMAPVAAAASQIRNTLVLLLSLNPMSLNASQLLPLNPPLYAARAGVDEFNHAQRVHVTPI
AN0239.1  	-----------------------------------------------------------M
          	                                                           :

MG02823.1 	AVNRPSRYRSSTSAST--------PPATSKAVEVLQSLFDQL~SQAADHRATPSGYPEIK
NCU00671.1	LAVRSRQYRSSTSAS---------AAQTSKAAEVLQSLLDRL~VATTNAPG-ANGYPDID
FG00191.1 	ALLELRQLRRSITEEGRGCLRHTCATPTSKADPPLSYSIEHS2IDTTLHQQ--DGFPQTP
AN0239.1  	NQAVSLSPHSSLTQSP--------FVEFAPVIDELKSICDDY~IDISLTGT---------
          	        : * : .             : .   *.   :      :             

MG02823.1 	KIVKHARELHQIVASSSPPCPAQDNFRHLRGFHRILDVLRAFSGFYNPQVRTEEEKKALF
NCU00671.1	ELIDHIRRIHQHIAAARPPSPPQDDFRHLHGFQTLLDVLRSFSGFYNPQKRTESERVGLF
FG00191.1 	PLLRKLRHIIQHISATRSP-VHQDDFRHADGFETILNLLRAFSGYYDPDKRTDADMLSLF
AN0239.1  	-VTECLLRLRHTLIDNPRPTEAKELFRQLSGFQTLLSLIRKLSEIYTPSVHTKEERRSLL
          	 :     .: : :     *   :: **:  **. :*.::* :*  * *. :*. :  .*:

MG02823.1 	ELLHIVLAVLSASFREHHGNRRYFRQRVEGGGWEALEQALASIGLGGSDADLWTNCQLFG
NCU00671.1	DLLHVILATISALFRGHPGNRRYFQDRVEGGGWEALEQIIASIGVGGGDSDLWTNCQLFG
FG00191.1 	KLIGDCLNLFSVALRGHAGNRRYFQYKVEGGGWEALEQVIASIGLGGAEPDAWISCHIFG
AN0239.1  	AVYKDCLTILAECLRDHLGNKRHFANRIPGGGQLVLEEAFSTLILK-LDAAQGDVEYFCG
          	 :    *  ::  :* * **:*:*  :: ***  .**: :::: :   :.       : *

MG02823.1 	KLLSFSLDDQRLHELCLWVGSSANTTTVTTGDNTKAENGTTDADSTGSSQAQTKGVEQPV
NCU00671.1	KLLSFSLDDQRLDELCRSVAEEGKALEQKP---TELES-NKQDEAQGQKEEVELQPTEAE
FG00191.1 	KLLAFSLDDEALDLLCQSIGQS-----------LRPDDENSPKEDESDSAEEQWDLVLAK
AN0239.1  	SVLAASLCQETVVDVFTALSTK-----------------------------LQKTDESEI
          	.:*: ** :: :  :   :. .                                      

MG02823.1 	APEIIQKKIDDIVGVKTVLHNAEILRAVVDFWESIPREKGSEANLASVLVLSTLTSVVKT
NCU00671.1	RWVIIQERLKAIIGSTTVIQNPEIIRTIVGFWESIPRAEGAAASHASMIVLTALSCVASV
FG00191.1 	SAENITPGVREVVNAKTIIRHPEILRAVVSFWTAIPRMKDGLANPASLLVLETIHCAISV
AN0239.1  	TPDAVEKEVGRFIGASDIIEAPELLGPFLRVWLKHSFLFKPVQTVLRLAVPSCLNRLATQ
          	    :   :  .:. . ::. .*:: ..: .*   .       .   : *   :    . 

MG02823.1 	SFFNLSALHSTGVLSRFLHLYFDPSSSLTQGEREKILPLCKSLMSLGVNNLADAQFLLTS
NCU00671.1	SFYNLTAIHGTGILSRLLRVLFSENSLLAEAEQKDAAWLCRKLISLGIDELADAQFLLSR
FG00191.1 	SIYNRAAVHSTGALSQFLRVAFNADSALSPPEREKVLAICKILMYLGVNEPADTQFLLST
AN0239.1  	SQRNVMILHNTGAFSLTLPLLLGEN--LLRTEKLFYQELAQQLCTQGTKNMDDAVYLYKK
          	*  *   :*.** :*  * : :. .  *   *:     :.: *   * .:  *: :* . 

MG02823.1 	RD--PAASEFCLEMTEKYDNPNFVQFDLSLHGHASIELPNLGRSFPPQSSPGYTFTAWVR
NCU00671.1	QD--AVMSEFCLDMSEKHNGPPFVQFDLSLHGHASIELPSLGRTFPPQSAPGYTFTAWVR
FG00191.1 	PG--SEASEFCLQMTNKHTGPPFFQFDLSLHGHSSLELSSLGRSFPPQSSAGYTFTAWIR
AN0239.1  	AHKCPKALKFLVDVLKRSKEPPLIQFDLSLYGFSSLEFATIGRSFPPVTTNGFTLAVWAR
          	  ...   :* ::: ::   * :.******:*.:*:*:..:**:*** :: *:*::.* *

MG02823.1 	IDKFDPNSHTTLFGIFDATQTCFLLAYLEKDTHNFILQTSVTSQRPSVRFKGYSFKPGQW
NCU00671.1	VDRFDPKSHTTLFGVFDSTQTCFLLAYLEKDTKNFILQTSVTSSRPSVRFKSVVFKPNRW
FG00191.1 	VDSFDPTAHTTIFGVFDSTQSCFVLMYLERDTHNFILQTSVTSNKPSVRFKSVSFKEKKW
AN0239.1  	FDHFDPSTHTTIFGAFDSSQTCFILAYLEKDTRNFILQTSIKGSRPSVRFKSMAFEPNKW
          	.* ***.:***:** **::*:**:* ***:**:*******:...:******.  *:  :*

MG02823.1 	YHIGLVHKRPKTMSASKAALYVNGEFVEQIRASYPSGPPLSNASTESFASFSSNANKTVP
NCU00671.1	YHIAIVHRKPKTLMTSKASLFVNGEFAEQIRSSYPSTPPLSNASTDSFASFTSNANKTNP
FG00191.1 	YHIAIVHRRPKTMVTSKASLYVDGEFSEQIRCNYPHLPPLATGAHDSFASFNSNQNKTNP
AN0239.1  	YHICVVHKKPKPPSHSRALLFVDGEFIEQQKIEYPCVP------------LPSAQNRSPR
          	*** :**::**.   *:* *:*:*** ** : .**  *            : *  *::  

MG02823.1 	VQAFLGTPRDLSTQVGPGLVFSRWSLASAHLFEDTLSDDFLAVHHRLGPRYEGNFQDCLG
NCU00671.1	VQAFLGTPRDLATQVGPGLVHSKWSLASAHLFEDVMSDDLLAVHYRLGPRYQGNFQDCLG
FG00191.1 	VQAFVGTPKDLSNQIGPGLVFSRWSLASAHLFEDALSDDYLAVHFGLGPRYQGNFQDSLG
AN0239.1  	VQAFFGTPRDLAMKLGRGVSSSRWSLANAILLGEAHSDDMIAVFYNLGPRYYGNFQDCLG
          	****.***:**: ::* *:  *:****.* *: :. *** :**.. ***** *****.**

MG02823.1 	GFQTYEASAALGLRNELLHPGKDENSDILRAIRGRASTILPESKVLLGSMPTAMFRTDDQ
NCU00671.1	SFQTYEASAALGLRNEIFHPGKDEVSDILRAIRDKAGTIVPEHKVLLSILPRAVFRSDGK
FG00191.1 	GFQTYEASATLGLRNEIAHPGKGETSDIIRAVREKAGSLLPESKILLSILPTATFPENVQ
AN0239.1  	SFQTYKASATLNLRNEHLHPGKEEQSDIVIAIRRKASTLVRESGILINVSARSILDDDDS
          	.****:***:*.****  **** * ***: *:* :*.::: *  :*:.  . : :  : .

MG02823.1 	--FLESRLFRSLSKVAANNLLAVTTKSGTAVAINTALPCVNDALVRAHGMSVLSGDPVLA
NCU00671.1	--FMESTLFRSLSRHSVTNLLHSTTKNGTPVAINGAVSCINDALIRANGSGVLTGDPVLA
FG00191.1 	--FYDTGLLRSLPRNCARNLFRASNQEGAPLAINCAVPSLPDSLFRVQGLLSFRGNPIVV
AN0239.1  	NAMDGSQLVKSLSKQARKLLNQFTKGGGNAIAINGASPAINDALIQPHGVGILTGDPVVT
          	.::  : *.:**.: .   *   :.  * .:*** * ..: *:*.: :*   : *:*::.

MG02823.1 	VPRNFDDSLWRTAGFKPVVLKLIERASTPEELVRIVELTFLCINKSWRNSDSMERDNGYG
NCU00671.1	TPYNFDDNLWRLAGFTPVALKIVERTTTGDDLVRAVEIMLHCITHSWRNSEAMERDN--G
FG00191.1 	VPSYLDENLWRLAGFTPLALKLFERASSIEETVRSAEMLFYCIRQSWRNSEAIEKDN--G
AN0239.1  	SPHSLDDMSWRIGGCASVHLSLIYSASSIESTLLAVEALYEAVQDSWRNSEVMEKEN--G
          	 *  :*:  ** .*  .: *.:.  ::: :. :  .*    .: .*****: :*::*  *

MG02823.1 	YAILAMLIRAKLGYGG--VDNPNWRLKMTAEERDRTSFQLLSLVLAFVGYKHADPVDSFI
NCU00671.1	YAILSMLLRAKLGYGVSVSDNPSWRLSLSPDEKTRLTFQLLSLVLSFVGYNHADPIESFI
FG00191.1 	YGILAMLLRVKLGYDRGSSDSAVTRLQVSNDDRDRLAFQLLSLILGFVGYNHVEPMESFI
AN0239.1  	YGILATILREKLGYGW--ASTKVAAVCSNDEQRSALSLDLLRLTLKFVGYDFEQPTRSII
          	*.**: ::* ****.    ..    :  . :::   :::** * * ****.. :*  *:*

MG02823.1 	INPLAYRVLLIDLNTFRKSSLITQELYYKQFVTFAVMSKYHQFNSRRLLRMR1IVKRLLD
NCU00671.1	VNPLAYRILLIDPDFWRKSAPMIQELYYKQFVTFAVNSKHHQFNSRRLLRMR1IIKRLLD
FG00191.1 	INPLAYRILLIDLDIWRKSAPRIQELYYKQFVTFAVKSKYHEFNSRRLIRMR1IVKRLLD
AN0239.1  	TNPLAYRVLLVDLEIWKYGAPSLVELYYSQFCVFARESQFRRFNSKRLARMR1VNKKLLE
          	 ******:**:* : :: .:    ****.** .**  *:.:.***:** *** : *:**:

MG02823.1 	AMKAESIQEETLPHFLSAFESLTKCNLSIEVHRSLALFITYAFHTSPSSQPRTPKPSSAT
NCU00671.1	ALKTETISEDILPHFLTSFEALIRCSYSAEVYRSLALFITYTFHTPANSLPRTPRALSSA
FG00191.1 	AMKGEVVSEDTVSHFMGAFEILVTSNFSQEVMRSLSLFITYAFHSQPLPVNRSPRALSIN
AN0239.1  	ALKGGSVSSQVLRPFVSAFRSLMESCLSADLLRSLALFITHALHKPKES-SGLQKKKSMR
          	*:*   :..: :  *: :*. *  .  * :: ***:****:::*.   .     :  *  

MG02823.1 	ATRSASPLVPVATRTAQRRYTG--ELVAPKTLTRKQLGVKILEMYAGLLCDQGGLAEIRK
NCU00671.1	SRSVTPGPGPYTAKRPSLEYSGPSSSNGSRMLTRKQLGVKMLEMYTRILCEKNNSVDISR
FG00191.1 	SRTGTPLP---RRRGTPTDPTAAGIASGLKFLTKKQLGVKVLSMYSRILCEKGNTTHIKK
AN0239.1  	FRTNSGRQGFPSNKGP--------------SMSTSQVATEMLRMLCSLVCDTRDLALIKK
          	    :        : .               :: .*:..::* *   ::*:  . . * :

MG02823.1 	FARAVTN-----------------K0WLLCLISEDDPQVVVLSSKILARLLVTHGVSYTS
NCU00671.1	FAKTVTNKVIDIKGSEKEFVLTSSQ~WLLYLLAENDPEIVVYGCKILARLLVTQPTSYTA
FG00191.1 	FARTVTN-----------------K0WLLYLLAEDDPEVVVHGCKILARLLVTHGSNYTS
AN0239.1  	FARTVTN-----------------K0WLLYLMCEDEPEIVVLASRILAKVLVTNGSTYNK
          	**::***                 : *** *:.*::*::** ..:***::***:  .*. 

MG02823.1 	KFGSKTGGFWIMAHRLKHWWDISTLWPICFAILFGKDVAQLDFSKPFDAATLLAMFGS--
NCU00671.1	KFASKTGGFWIMAHRLKHWWDIPTLWAILFSILFGYDVAQIDFDKSFDFFSLLEIFGD--
FG00191.1 	KFAGKSGGFVIMSNRLKRFWDMPTLWPICFSILFGYDVAEINFDRNFDFFNILETFGK--
AN0239.1  	RFKEKNNGYIIMRGCLRRWWNIPILWPLCFSVLFGIDLGTTMADRLFDNASLCRVLMANE
          	:*  *..*: **   *:::*::. **.: *::*** *:.    .: **  .:   :  ..

MG02823.1 	GRVSLPDVLPVIMSMLQHGLRDVMKYQDDPDSPVSSFEADRT-----AKIQPQNGHEVQD
NCU00671.1	SKVVYPDVLPVMTAMLQHGLRDVLRYQDDPDSPAAEHSGATTSGGNLAAVHTRPRARSME
FG00191.1 	RKVVYPESLSVITAMLQHGLKDVMRHQDDPDSPATYVDPASH--------TLKKFASAKD
AN0239.1  	VKVCFPEIFPVIADMMRSALRKSVMAHGSLHG----------------------------
          	 :*  *: :.*:  *:: .*:. :  :.. ..                            

MG02823.1 	TADKEGGRPRARSMALADELASRQTFR----PDQERVNTHSAVLYIVIRFLSDLHTRSSD
NCU00671.1	LGQALESRSKLDPAFSSSIVATNERFPGAPMPDKERVVNHASILQTVVRFMADMYNRSES
FG00191.1 	LRP--EMRPRASSMNLTTALATRSNFS----RDSERVASYAGVLHTVIRFLLDLHERSTE
AN0239.1  	----LDLDQTARPLMAHPVPVDTNDQE-------------ALLLATVIEFLAGLHLKSEG
          	            .       .  .                : :*  *:.*: .:: :*  

MG02823.1 	FRDFCLGSDYVKMILSALYPILLSTDPVNPETELNSKDSALTFEGGDVIIRPVS-GSGSA
NCU00671.1	FRDFALASDYVRLLLSALYPIVVSADPVSPETELGSKDSSLNFDGIDVIIRPVVRSSSSA
FG00191.1 	FRDFALTSEWVRLLLSALYPVIVSADAVTPETELNSGDTALTFEGTDVIIRPISGSGSHA
AN0239.1  	FREFASKSEYAEDVLSVLFPVVVGSDSVSADIELSYRVSGLGLDDHNSRASPPQ---SRN
          	**:*.  *::.. :**.*:*:::.:*.*..: **.   :.* ::. :    *     .  

MG02823.1 	APIVRTTTASLLDRPSAAPLQTAKGTPLRKASSFVLLTSSKSPQLPSSARFNPIMSPKQK
NCU00671.1	TPIVRTTNTAVAEQPNSSTGQ-SRGTPLRRPSSFILVAQDQ-PMSPPPSRLNHVMSPKKK
FG00191.1 	TPIVRTTSVDPVPSPQSTPPK---GTPLRRASSFVLLTQQKSPVGPSTARLSPALPSPRS
AN0239.1  	TNELHTTTVEHSGTRDETSSS------LPRSSSFILVSQHGGKQSRSPLQIKHSFALGSM
          	:  ::**..      . :. .      * :.***:*::.  .    .. ::.  :.    

MG02823.1 	VVTQKVLNMVFDGLLDLVVKVFADQIFARKEFQGFALSSKIPPGFQEHQAYLESYILRST
NCU00671.1	VATQQVSNVVLESILELIISVFNDQILSRKDFTGFGLFLKVPPGFQEHQAYFETYILRNA
FG00191.1 	TPSKRPSSDILDGLMELVVSVFMDQLLSRKDFSGFGLFTKVPPGFREHQAYFESYILKHV
AN0239.1  	DIEGLTEHPFLSRILCLILSIFSEQLLERKDFSGLGLFSKTPPGFLEHQAYFNSWVLTKL
          	         .:. :: *::.:* :*:: **:* *:.*  * **** *****:::::*   

MG02823.1 	ISHLDTTLQKDQKLLCEPKILVNMSRFTVHVVEAIFEGWFLNGAEMMLDFAGTMLEYLQR
NCU00671.1	ITQLRNAVQLDQKALLEPRVLQNMSRLNMHMAEAIFEGWFINGAETMLDFTGTLLEYLQR
FG00191.1 	VAQLEKNIQLNQKAICEPRVLTNLSRLCQHMSEAIFEGWFLNGAEVMIDFIGMLLEFLQR
AN0239.1  	LQMLKDVAFSKTELLAEPRVITNLGRFTTHLAEATLEGWFIDGAMATLEFSGTILEYLQR
          	:  *      . : : **::: *:.*:  *: ** :****::**   ::* * :**:***

MG02823.1 	PDISSLKSVRLCSQPVNTIRHSFLRLALLRLTEMGDPQMSDAEALSVMKNIFYWQTVLLS
NCU00671.1	PDVSSTKSVRLCSSAVNTIRTTFLKLVLLKLSDMDDPQTTDQEATSIMEKLLYWQAVLLN
FG00191.1 	PDIASLKSVRLCSQAVNSIRFCLLRIILLRLSDLDSSDTTLVEAKQFMGKMAYWQMSILG
AN0239.1  	PDVSSLKSIRLCSQIIATIRSNLFRVVLVKLSEVQGP-----DALSFLNCLSYWQVVLLQ
          	**::* **:****. : :**  :::: *::*::: ..     :* ..:  : ***  :* 

MG02823.1 	SVSSEDDYIKLLWYQLYAKLVDSREQIRLVAASVWRIMLVQKPDESASILRQCATIEQQP
NCU00671.1	CLSLDDDFMKLLWYQFYNKLVDTREPIRLMAAMLWRIMLVQKPEESSMLFRQFMTPDQQP
FG00191.1 	SFTGEDQFLKLFWYQLYTKLISDKMPLRTAAANFLRVILVQKPEESNELIHSCMSPDQKQ
AN0239.1  	AGESQADNLQLLCYLLYTKLIDENKDVRVAAAGLFRVIMVQKPAELFVILNHAAVPLQER
          	.   : : ::*: * :* **:. .  :*  ** . *:::**** *   ::.      *: 

MG02823.1 	LVKNFCKLTEVDDGNFVKWIDEHRPSLDLLFFGGMSRPWEEYVAVENQRTMDTARSRLSR
NCU00671.1	LAKGFRRLTQLDDVSFLEWVDSYRSSLDILFFGGMSKAWEDYVRAENQRTADTARSRMRN
FG00191.1 	LSKDFQQLTGVDDESFVEWVDKHRPSLDVLFFGGMSKTWEDFVSSENARTAETSKVRLAK
AN0239.1  	LSGGFEALAGMDDAAFLEWIDNHADDLNTLFLGNVSKSWEDFIHQQNTAIETSWRIRMTK
          	*  .*  *: :**  *::*:*.:  .*: **:*.:*:.**:::  :*     : : *: .

MG02823.1 	RREKLRQWHAEGLERDNVLLRHDMSNGAWAKSIYLNEHHRHQRLMQDQQDDLAFHASAFA
NCU00671.1	RKDKLRQWHTEAMERDSILLRHEMSNNAWMKSIYFTEHFKHQRLQQDQQDDNAFLASSFA
FG00191.1 	RKDKLRVWHAENINTEKTLLSHDIGNSAWMKSIYNSEYFKYQRIMQDQQDDLVFLSDAYK
AN0239.1  	RHERLKQISQMEKARDEVTRRHEVTFPHWSSNIANSELLRYHRALQDQQDSHVFMWNEFF
          	*:::*:         :.    *::    * ..*  .*  :::*  *****. .*  . : 

MG02823.1 	KMDRDLRRPGAMFAEPTTIKWKLDRTEGRNRMRLRVLPDYTPQQ-PEYQTKQRRNTGSGP
NCU00671.1	KMERELYRPGAVFAKEQKIKWKLDRTEGRNRMRLRLLPDYAAQQQPDYQP--RGNAGSG-
FG00191.1 	KMERDLMRPGAVFSEAAPPKWKLDRTEGRNRMRLRVLPDFTISK-DEYQP--KRKASEV-
AN0239.1  	RLTLDLRRFGGILAEEKERKWRLDQTEGRSRMRLRIVPDDTKER-QDYQP--KRKASEP-
          	::  :* * *.::::    **:**:****.*****::** : .:  :**.  : ::..  

MG02823.1 	QPNITQSPTPGLKLNTDVNLVVHPPSAGGIGSTPLPPEPGQGPDKSKDFDFAAEADAIPE
NCU00671.1	------APASSLAAGDSSSSGRRSRAP----SSVAQPQSKAGAEPSSDSNTNLAPKFAPG
FG00191.1 	--------LQTLRLNTSASPAPSIVTS---AVSPTKTATPALPESNNETPQFTEEPESTV
AN0239.1  	---------PVVKVDASGPSNGKTLAP------------PLDDERSTDSSHGIGPDNAS-
          	           :  . .        :.                : ..:          . 

MG02823.1 	EDADPSKGLGPEDDFEVIENP----EPDDDFEDKQRKVMRRLEHGDQVRSVYNISRIIGL
NCU00671.1	DSDQQS--VSPEDDYELVDDPNE--QEDDAFEDKNRKVMRRLQQGDTVQNVYNISRIIGL
FG00191.1 	PAEQPTSGVAPEDDFELVDDPNDPNEGDEGFEDKNRKVMRRLERGDQVQSAYNISRIIGL
AN0239.1  	---------VMEESFEMISDP---AADLEDYDDKNRKVMRSLHRGDEVENLCNVSRIVGL
          	           *:.:*::.:*       : ::**:***** *.:** *..  *:***:**

MG02823.1 	DACEGILLIGKDALYLMDNIFQRADGEIVNVWLAPPEERDPFSQIITQDKPPEKRQTSKR
NCU00671.1	DASEGILIIGKEALYLMDNLFQSPDGEIINVWQAPPEERDPFSVMIS-GQSNDRRQGQNR
FG00191.1 	EACEGILIIGKDALYIMDNVFQCANGDIVNVWQAPPEERDPFSQIVTDAKTSEKRQNNK-
AN0239.1  	EAFEGLLIQGKDHIYILDNFFQRVDGEIVNVSQAPSDERDPYVRMIAGREASER----KP
          	:* **:*: **: :*::**.**  :*:*:**  **.:****:  :::  :. ::    : 

MG02823.1 	SEQESRHWRWSDVISISKRRFLFRDVAIEIFFRDGRSYLLTAINPVIRDEIHGKLLQKAP
NCU00671.1	TDQESRSWRWQDVLSVSKRRFLFRDVAIEIFFTDGRSYLFTALNSAMRDEIYSRMMNKTP
FG00191.1 	-EQESRHWRWHDVISISKRRFLFRDVAIEVFFTDGRSYLLTTINPVVRDNLFIKMLSKAP
AN0239.1  	QEYETRSWKWTDLVSISKRRFLFRDVALEIFFTDGSSYLLTLISSKARDILCSQLAGKAP
          	 : *:* *:* *::*:***********:*:** ** ***:* :..  ** :  ::  *:*

MG02823.1 	HTTGPNSLPNPEDAWRLEALRVYEEAPQTFGSKFGSIFNSSPWNPIMKRWQKGEISNFHY
NCU00671.1	HSNGPNLLPTPEDAWRLESLKFSEEAPQTLGMKFGSIFNSSAGNPMMRKWQKGEISNFHY
FG00191.1 	HTTGANTLPNPEDAWRLESLKVFDESPQGFGSKFGTFFNASPWNPILKKWQKGEISNFHY
AN0239.1  	QVTGNVGHSRPEDIWRFETLRSSEDAPQSLGSKFASVFGHSPVYPATRKWVKGEISNFHY
          	: .*    . *** **:*:*:  :::** :* **.:.*. *.  *  ::* *********

MG02823.1 	LMLVNTMAGRTFNDLTQYPVFPWVLADYTSEELDLNDPATFRDLSKPMGAQTLSRQSDFR
NCU00671.1	LMLVNTMAGRTFNDLTQYPVFPWVLADYTSEELDLNDPATFRDLSKPMGAQTKSRMDTFM
FG00191.1 	LMMVNTMAGRTFNDLTQYPVFPWVLADYTSEDLDLDDPNTFRDLSKPMGAQTPHRVGGFV
AN0239.1  	LMLINTLAGRTFNDLTQYPVFPWVIADYTSQELDLTNMKSFRDLSKPMGCQTPEREADFR
          	**::**:*****************:*****::*** :  :*********.**  *   * 

MG02823.1 	ERYNGLAELEL---PPFHYGTHYSSAMAVASYLIRLPPFVDSYLLLQGGHFDHPDRLFYS
NCU00671.1	AKYQSLAEMDE---QPYHYGTHYSSAMIVSSYLIRLPPFVQTFILMQGGSFDHADRLFYS
FG00191.1 	ESYNALEEIGE---VPFHYGTHYSSAMIVSSYLIRLPPFVQSYILLQGGSFDHADRLFQS
AN0239.1  	ERYQAFAEMGDGDSPPFHYGTHYSSAMIVSSYLIRLQPFVKSYLLLQGGTFDHADRLFYS
          	  *:.: *:  ..: *:********** *:****** ***.:::*:*** ***.**** *

MG02823.1 	VAGAWKSASQDNGSDVRELIPEFFCLPEFLTNINGYDFGMRQ-DGSRVSDVILPPWAKGD
NCU00671.1	IEGAWASASRDNGTDVRELTPEFFYLPEFLTNINGYNFGTRQGDGGKVDNVILPPWAKGD
FG00191.1 	IPEAWQSASCKNKTDVRELIPEFYCLPEFLTNVNGYNFGNRESNGVKVDHVELPPWAKGD
AN0239.1  	VGKAWESASRGNMSDVRELIPEFFYLPEFLVNSNKYDFGVLQNMATTIDSVELPPWAKGD
          	:  ** ***  * :***** ***: *****.* * *:**  :. .  :. * ********

MG02823.1 	PRIFIARHREALESPYVSQQLHRWVDLIFGYQQRGDAAVENLNVFHNMSYHGAIDLDGIE
NCU00671.1	PKIFIAKNREALESPYVSQRLHQWIDLIFGYKQRGDAAVEACNVFHPLSYKGGKDLDNIS
FG00191.1 	PRIFIAKHREALESPYISENLHQWIDLVFGFKQRGEAAVENLNVFHHLSYRGATDLDSIT
AN0239.1  	PKIFIAKNREALESPYVTRNLHHWIDLVFGCKQKGEAAVEAVNVFHHLSYQGAKDLDAIN
          	*:****::********::..**:*:**:** :*:*:****  **** :**:*. *** * 

MG02823.1 	DPQERKVITSIIHNFGQTPHQIFSKPHPSRENVHCQIKRLDTSIFALTKLPYPLL-----
NCU00671.1	DPQERLIATNIIHNFGQTPHQVFTKPHPAREHARCPIKRLDTSVHSLTNQPHPLL-----
FG00191.1 	DAKERAILAGVIHNFGQTPHQVFMKSHPAREHVRSPARRLDTSVFSLSCLPHPLLGMDSP
AN0239.1  	DPVERLATIGIIHNFGQTPHQIFNRPHPQSEVHKHKTLRLDTLAESLTQLPIALL-----
          	*. **    .:**********:* :.**  *  :    ****   :*:  * .**     

MG02823.1 	------------E1SHERVASLIYAPKLDRLLCASPFRVNLPPHYDKFLEWGYADNSVR2
NCU00671.1	------------D~SHERVSSLIYAPKLDRLLCASPFRLNLPPQYDKFLEWGYADGSVR~
FG00191.1 	SRSMNLTDKPSIE~SHERVASLLYASKLDRLLCASPFRLNFP-SYDKFLEWGYADNSIR~
AN0239.1  	------------E1TGERVSSLCMRN--DRLLCTAALRLNIPPNYDKYLEWGFFDGSVR~
          	            : : ***:**      *****::.:*:*:*. ***:****: *.*:* 

MG02823.1 	SSKKKKA------------KDKNAK~LAGIFENLHIGQISCVAFADSKTLITAGEDGVIS
NCU00671.1	FFFTN-----------------DRK~PAGLFENLHIGQISTLTFADSKTLITAGEDCVVS
FG00191.1 	FFFSDNRKVIRNHILVHGRTANSTK~PAGLFENLHTGQISCACFADSKTLITAGEDCVVS
AN0239.1  	FYSTD-----------------NRK0LLGHFEHLHVGQLTAAAFADSRTLVTSGADCTVS
          	   ..                 . *   * **:** **::   ****:**:*:* * .:*

MG02823.1 	AFTIQTSPGKPVELLPRSSMFGHKSPVTTLAVSKSFSTLVTVARDGEAFLWDLNRLEFIR
NCU00671.1	VYTVQSSPGKPVELVPRSSLFGHKTPVTTIAVSKAFSTFVTVSQDGISFLWDLNRLEFIR
FG00191.1 	VYALQTAHGKPVELLPRSSLFGHKFPVTTIAVSKAFSTLLTVSTDGQAFLWDLNRLSFIR
AN0239.1  	IWTFTAN-SRSVDLQPAGSLFGHRTPVTVLAVSRSFSTLLSASIDGQLMLWDLNRQCFLR
          	 ::. :  .:.*:* * .*:***: ***.:***::***:::.: **  :******  *:*

MG02823.1 	KLKHYGRRPVE~CARVNDVSGEIMLCSGTNVMLFSVNGDLLVDQNVCGTNTSNSRDDYVH
NCU00671.1	KLP--LARPVE~CARIHDVTGEIMLCSGPNVLLYTLNGELILDQNVCSEGDTN---DYIH
FG00191.1 	KLP--LVRPVE~SARINDISGEILLCSGPNVILYTLNGTLLLEQNVCFD-----QDDYIH
AN0239.1  	ELP--LKGPVD0VVCESS-------------------------------------DDTTL
          	:*      **:  .   .                                     .*   

MG02823.1 	SCAFYEGS-------GGNEWLENQLVFTGHRRGKVN~IWRRVVSP---------KTGKWE
NCU00671.1	SCAFYEGTSTSTVGGGGNEWVEENLVFTGHKRGRVN~VWRKTVAEYKKGSASGGSSSRWV
FG00191.1 	SCAFYEGA--------GNEWLDNYLIFTGHKRGIVN~VWRRSIVG-----------SRWT
AN0239.1  	ACVFYEGV--------GDEWQERELVFTGHKRGVVN0VWSKIIRN-----------GRFE
          	:*.****         *:** :. *:****:** ** :* : :             .:: 

MG02823.1 	LEFLRRLDHIDTKSETGANVEAAITCIAPMPQVVYTGDEEGRV0YEWNLVQRER------
NCU00671.1	LELVRRLDHINTKSETGQNVEAAITCITPMPSCVYTGDDDGRV0YEWNLIQRER------
FG00191.1 	LELLRKLDHVDYKNEKGANTEAGMTCISPMPTCVYTGDDDGRV0IESGPPEPDISSTQPP
AN0239.1  	LELIRQLHHIDNNRDNGANIAAGISCIQAFPQVVYTGDEAGRV0AAGGYSSLNGSHCRRD
          	**::*:*.*:: : :.* *  *.::** .:*  *****: ***    .  . : :     

MG02823.1 	---------------------------------
NCU00671.1	---------------------------------
FG00191.1 	Q--------------------------------
AN0239.1  	YGYTTPLYGVQRSYRKGKPYFCYSGALGLDTSP
          	 . ::.  . . :  ....  . :.: . .::.
```
